# A New Controller for a Smart Walker Based on Human-Robot Formation

**DOI:** 10.3390/s16071116

**Published:** 2016-07-19

**Authors:** Carlos Valadão, Eliete Caldeira, Teodiano Bastos-Filho, Anselmo Frizera-Neto, Ricardo Carelli

**Affiliations:** 1 Postgraduate Program in Electrical Engineering, Federal University of Espirito Santo (UFES), Fernando Ferrari Av., 514, 29075-910 Vitoria, Brazil; teodiano.bastos@ufes.br (T.B.-F.); anselmo@ele.ufes.br (A.F.-N.); 2 Electrical Engineering Department, Federal University of Espirito Santo (UFES), Fernando Ferrari Av., 514, 29075-910 Vitoria, Brazil; eliete@ele.ufes.br; 3 Institute of Automatics, National University of San Juan (UNSJ), San Martín Av. (Oeste), 1109, J5400ARL San Juan, Argentina; rcarelli@inaut.unsj.edu.ar; 4 Consejo Nacional de Investigaciones Científicas y Técnicas (CONICET), C1425FQB Buenos Aires, Argentina

**Keywords:** smart walker, robotic walker, accessibility, mobility, assistive technology

## Abstract

This paper presents the development of a smart walker that uses a formation controller in its displacements. Encoders, a laser range finder and ultrasound are the sensors used in the walker. The control actions are based on the user (human) location, who is the actual formation leader. There is neither a sensor attached to the user’s body nor force sensors attached to the arm supports of the walker, and thus, the control algorithm projects the measurements taken from the laser sensor into the user reference and, then, calculates the linear and angular walker’s velocity to keep the formation (distance and angle) in relation to the user. An algorithm was developed to detect the user’s legs, whose distances from the laser sensor provide the information necessary to the controller. The controller was theoretically analyzed regarding its stability, simulated and validated with real users, showing accurate performance in all experiments. In addition, safety rules are used to check both the user and the device conditions, in order to guarantee that the user will not have any risks when using the smart walker. The applicability of this device is for helping people with lower limb mobility impairments.

## 1. Introduction

Mobility can be defined as the “ability to move or be moved freely and easily” [1], which is an important skill of the human body that affects virtually all areas of a person’s life, since it is used for working, entertaining, having social relationships and exercising, among other daily tasks. Mobility also works as a form of primary exercise for the elderly [2]. People whose mobility has been impaired usually rely on other people having to perform daily tasks. In addition, the lack of mobility, inevitably, decreases the quality of life of those affected [3].

Impaired people can lack totally or partially the force needed to perform their movements. In the first group, the affected limb is unable to perform any force to allow the movement, while in the second group, the force is not enough to perform the movement [4]. Having a place or device in which the person can support partially his/her weight helps with balance and makes it easier to walk.

Assistive technologies (AT) are used to help impaired people, including those with problems related to mobility. They are defined as a set of equipment, services, strategies and practices, whose concept and application are used to minimize the problems of people with impairments [5]. This kind of technology has gained importance and awareness, due to the increase of the population who need assistance in several forms, including mobility, especially for the elderly, who can suffer from fall-related problems [6].

Mobility-impaired people may resort to some assistive devices to improve their quality of life, independence, self-esteem and avoid the fear of falling [7,8]. Several situations may lead a person to require an assistive device, such as infirmities, accidents and natural aging, which brings some age-related illnesses [9], and studies from the United Nations show that there is a tendency of the increase of the elderly’s participation in the overall population [10]. This shows the importance of studying technologies to assist the elderly. In addition, there are also people with non-age-related illnesses that affect mobility.

The mobility-aid devices are divided into two major categories: alternative and augmentative ones [7]. In the first category, the device changes the way the person moves himself/herself and does not require residual force of the affected limbs to allow the movement [7]. Examples of this category are wheelchairs, cars adapted to elderly people [11] and auto-guided vehicles (AGV), which allow the user to move without using the force of the affected limb [12]. By using these kinds of auxiliary devices, people who do not have or are unable to use remaining forces can benefit themselves [13,14]. In contrast, in the second category, the augmentative devices are designed for those who can still use their residual forces to move. These devices only work if the user intentionally applies the remaining forces to aid the movement [15]. Examples of these devices are canes, crutches, walkers and exoskeletons [9].

Each group has its advantages and disadvantages. Remarkably, the alternative devices can be used for most kinds of impairments, i.e., for both people with and without residual forces; however, the disadvantage is exactly the lack of the use of residual forces, because if a person still has remaining muscle forces and does not use them, his/her muscle may suffer atrophy [16,17,18]. Thus, augmentative devices use these remaining forces, therefore keeping the muscle tone and avoiding atrophy. The disadvantage of these devices lies in the fact that not everyone who suffers from an impairment can use them, exactly because they need residual forces, and some people may not have them [7].

**Figure 1:**
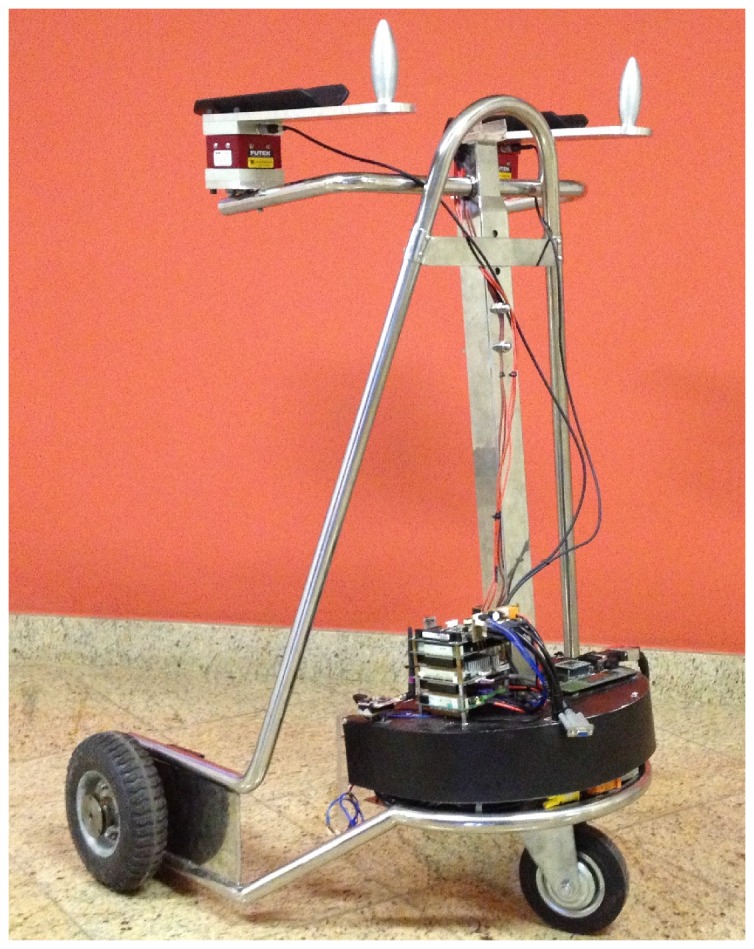
UFES’s smart walker.

The choice of the mobility-aid device must be made by a physician, who will take into consideration all of the factors and issues about the user’s health and rehabilitation needs [9]. If there is still usable remaining forces in the affected limbs, an augmentative device must be chosen [19,20]. Additionally, augmentative devices can be used as tools for physiotherapy sessions to enhance the muscular tone of the weakened limbs and improve the movement ability [21]. Some authors make a subdivision of augmentative devices according to the application: for transportation and for rehabilitation (for both transportation and physiotherapy) [7]. Figure 1 exemplifies this kind of device, which is a smart walker developed in our lab at UFES/Brazil to help people in gait rehabilitation [22].

**Figure 2:**
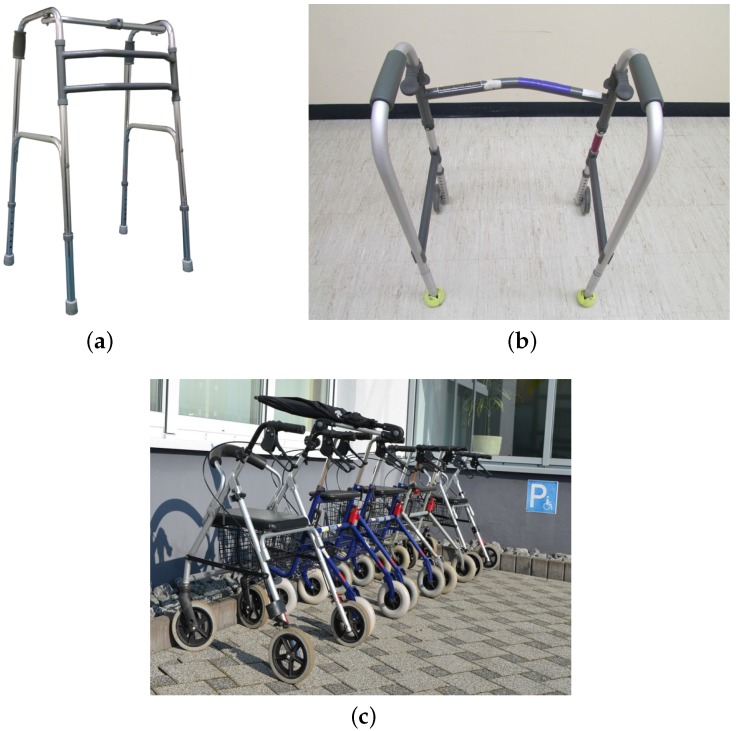
Types of walkers according to their mechanical structure. (**a**) Four-legged; (**b**) front-wheeled [24]; (**c**) rollator [24].

In terms of the number of frames, walkers are categorized into basically three types, according to their ground reaction forces [23]: four-legged, front-wheeled and rollators (Figure 2). Each one has its advantages and disadvantages, which are intrinsic to their construction.

Four legged walkers are considered the most stable of the conventional walkers and can be used for people who do not have good body balance [23]. As a disadvantage, this kind of walker requires more force to use it, since the user has to lift the whole device and then put it back on the ground at each step. Nevertheless, this walker does not offer a natural gait, due to the need for lifting it [7,9,25]. On the other hand, front wheeled walkers do not require much force, since the user has only to lift the walker in the rear part, keeping the wheels on the ground and using them to move the walker. This walker provides a better gait when compared to the four-legged one; however, it still requires lifting the walker up, although not completely, during the gait, requiring less force than the previous one, but offering less stability and requiring more balance and control, especially when the walker is not totally on the ground [7]. Finally, the third type of walker is the rollator, which is the one that provides the most natural gait. In addition, it requires less force than the two previous walkers, since no lifting is necessary. However, this structure requires from the user better control and a good balance, since the wheels can run freely [7,9,23,25].

The rollator is the kind of walker used in this work, although built from a four-legged frame (converted to free wheels) and attached to a mobile robot to become a smart walker, with electronics, sensors, control system and actuators (motors) added to it.

### 1.1. Smart Walkers

As previously mentioned, smart walkers are walkers that contain, besides the mechanical structure to support the user, electronics, control systems and sensors, in order to allow a better user experience and minimize the risks of falling. In addition, they also provide a more natural and soft gait [7,26] as they are usually built on a rollator structure [25]. Some of them are designed to help users in other functions, such as guiding visually-impaired people or elderly people who have memory deficits [27,28,29,30]. There are also examples of walkers that go beyond the mobility support and expand the user experience, providing sensorial, cognitive and health monitoring support [7]. They can be classified into passive or active depending on the propulsion movement being made/aided by the device or not. A passive smart walker may contain actuators to help the orientation of the device, but not propulsion, while an active smart walker provides support in propelling the movement [15]. Some notable smart walkers that contain other features, besides aiding the users with movement, are:

- RT Walker: this is a passive walker, which contains a laser sensor to monitor the surrounding area and two other laser sensors to find the user’s legs. In addition, this walker also contains inclinometers to find the walker inclination [31].
- GUIDO Smart Walker: this walker had its first version (PAM-AID walker) developed to assist blind people [32]. Throughout time, this walker received new sensors, other control strategies and, currently, has ultrasound or a laser sensor (depending on the version) to help users avoid obstacles [29]. They do not offer propulsion, being classified as a passive walker [33]. The current version of this walker can also perform SLAM (simultaneous localization and mapping), which allows the device with mapping the environment (while assisting the user) and using this information to detect obstacles [27].
- JARoW: this walker uses omni-directional wheels to improve its maneuverability. Two infrared sensors are used to detect the user’s legs in order to control the walker speed [34].
- Other devices: there are several other devices, with different techniques and purposes. The SIMBIOSIS Smart Walker, for example, was designed to study human gait [35]; the PAMM (Personal Assistant for Mobility and Monitoring) monitors the user’s health while he/she uses the device [36]; the iWalker was developed to be used inside a structured environment using local sensors and others in the environment [30]. These walkers are focused either on elderly people or to study human gait.

In this work, a new controller for a human-robot formation is introduced, in which the human is the leader of the formation and does not have any sensor on him/her, and the follower is the robot, which contains all of the sensing devices. All measurements necessary for the walker controller are obtained from the distance to the user’s legs (through a laser sensor), in addition to measurements from the ultrasound sensors and robot odometry. No force sensors are used, which is an advance in relation to the smart walker developed in [22].

Table 1 shows the comparison among the smart walker controllers. As can be seen, the smart walker of this paper proposes a novel controller based on human-robot formation. Besides, this work does not have any sensors attached to the user’s body, as some of those presented in Table 1 have.

## 2. Materials and Methods

### 2.1. Mechanical Structure and Hardware

**Figure 3:**
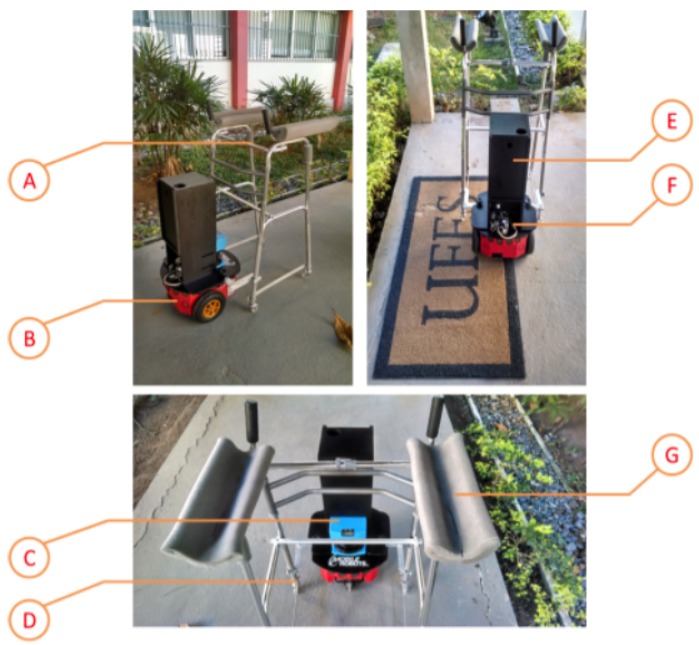
Diagram showing the structures that compose our smart walker [39].

Our smart walker was designed by adapting a conventional commercial four-legged walker (Figure 2a) into a structure, which was attached to a Pioneer 3-DX robot (Figure 3); the original four-legged walker was modified to include free wheels. Thus, our smart walker can move freely in any direction, and once attached to the robot, its movements are given by the robot. The human, in turn, controls the robot. Therefore, the movement is guided by the human.

To make the whole structure of the walker, some pieces were built in aluminum to attach the walker frame to the robot. Figure 3 shows details of the smart walker. Item A is the modified walker with four free wheels (Item D) and with other options for height configuration. Foam supports for the forearms were built to give more comfort to the user during the use, shown in Item G. Item B shows the robot and the ultrasound sensors to detect obstacles. The robot provides both propulsion and the non-holonomic restrictions needed for this application. Items E and F are, respectively, a support to store the battery used by the laser sensor SICK LMS-200 LRF (item C, [40]) and a WiFi to Ethernet converter used to allow the robot to communicate wirelessly.

### 2.2. Algorithms

The controller used as a basis to develop the human-robot formation controller presented in this paper was based on the studies of [41]. Here, the idea is to substitute the master robot (leader) with a human, with all of the sensors included in the follower robot. The human does not have any sensors, thus the acquired data by the robot are translated and rotated and then projected into the human reference. In other words, the data collected by the robot are processed and projected on the human pose as if the human were the master robot. Thus, the human commands the formation. By using the laser range sensor, the smart walker can calculate the human speed, position and orientation inferred from the distance to the human’s legs and, therefore, adjust its own speed, keeping the formation maintained. After the output of the formation control, the data of the linear and angular speed are processed by an internal servo PID (proportional-integral-derivative) controller, which calculates the rotational speed of each wheel of the robot.

**Figure 4:**
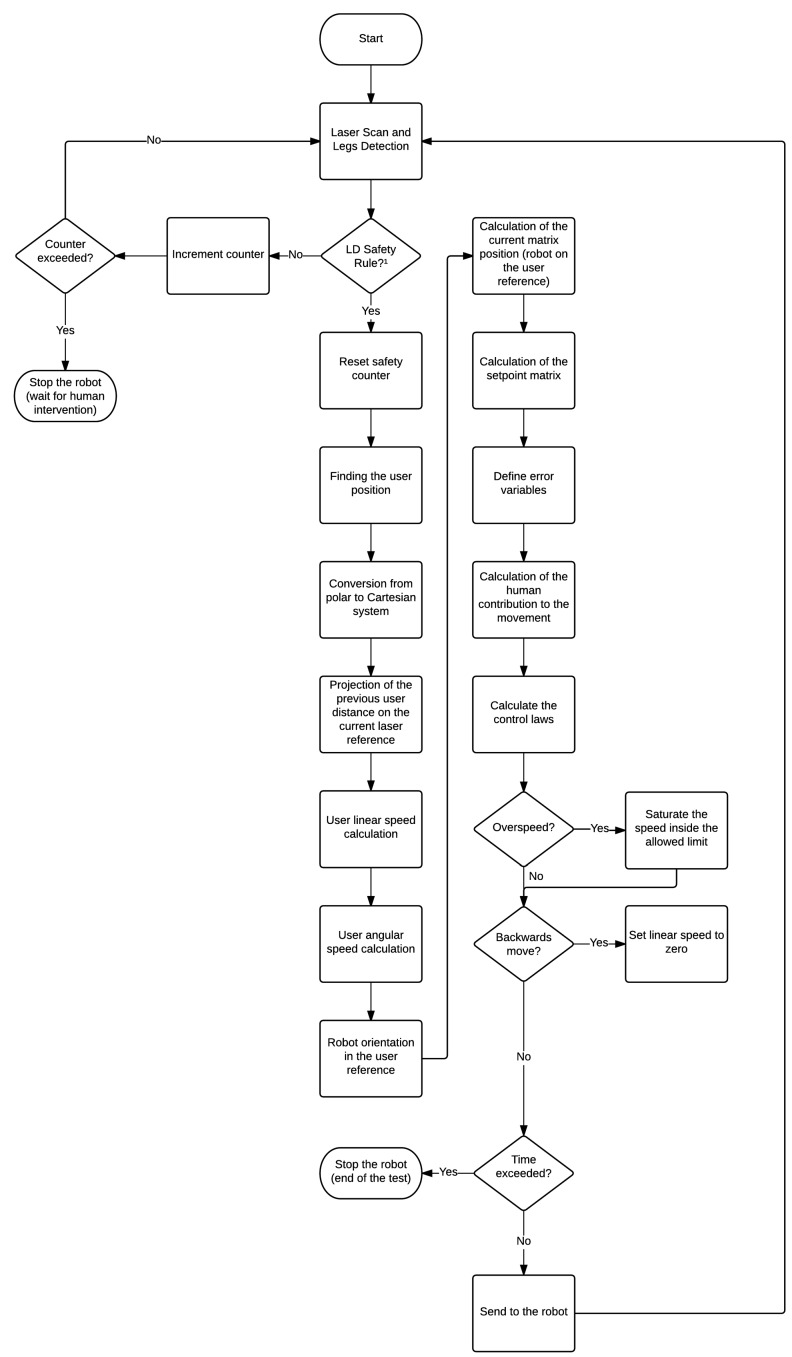
Flowchart of the data acquisition, controller and safety rules applied to the smart walker.

The steps of the controller are presented in the block diagram shown in Figure 4. Blocks, such as “LD Safety Rules” (LD meaning leg detection), “Overspeed” and “Backwards move”, shown in Figure 4, are related to the safety rules, explained in Section 2.2.4. The human-robot kinematics is explained in Section 2.2.2.

#### 2.2.1. Legs and User Detection

To detect the human pose, the laser sensor firstly scans the area in front of it (180°) with 1° resolution, searching for the legs [40]. The control algorithm uses only the filtered data in a region of interest (ROI), defined from 68° to 112° and 1 m long, which is the area where the human’s legs should be located. Inside this region, only the human’s legs should be detected, as the walker frames are outside that region.

**Figure 5:**
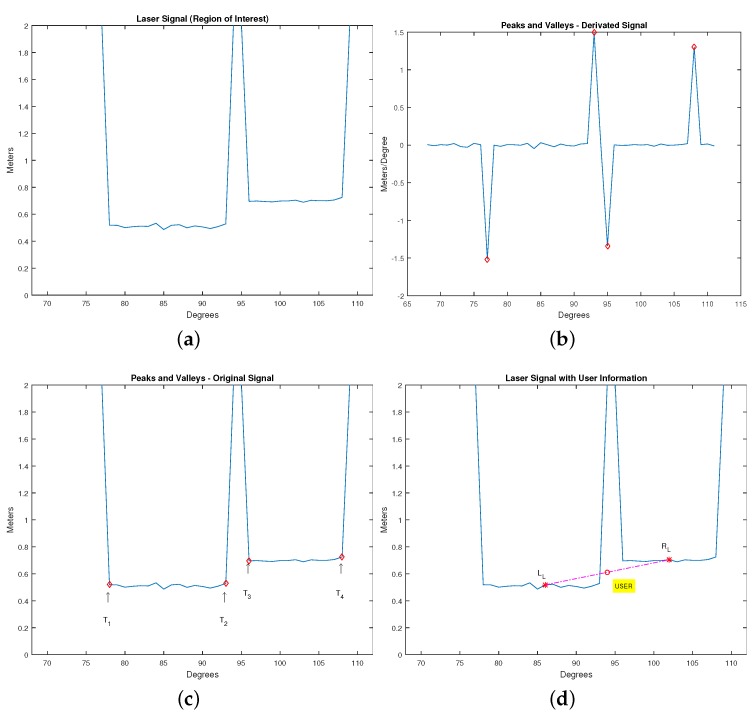
Example of finding the user based on the position of each leg. (**a**) Laser signal inside the region of interest; (**b**) signal derivative and transitions (peaks and valleys); (**c**) transitions in the original signal; (**d**) legs and user position.

The algorithm to detect the legs is based on the one developed by [42]. This algorithm works with the signal transitions, which are high variations in the laser signal. Transitions are actually big variations in the laser signal, and they can be found by deriving the main signal and finding $\frac{d\rho }{d\theta }$. By analyzing this last signal and finding the peaks and valleys, it is possible to infer where the transitions provoked by the legs are. Figure 5 shows in pictures what the transitions are and how they are analyzed (algorithm functioning).

The variables $T_{1}$ up to $T_{4}$ represent the transitions (high variation of the laser signal), and $L_{L}$ and $R_{L}$ are the left and right leg, respectively. Finally, the variable “user” means the position of the user itself.

Summarizing, the algorithm detects where the legs are located by performing the analysis of the number, length and amplitude of the transitions in the derivative function of the laser signal inside the ROI area. These transitions express big variations in the laser reading, generating peaks and valleys, which are used to find the legs’ extremities.

**Figure 6:**
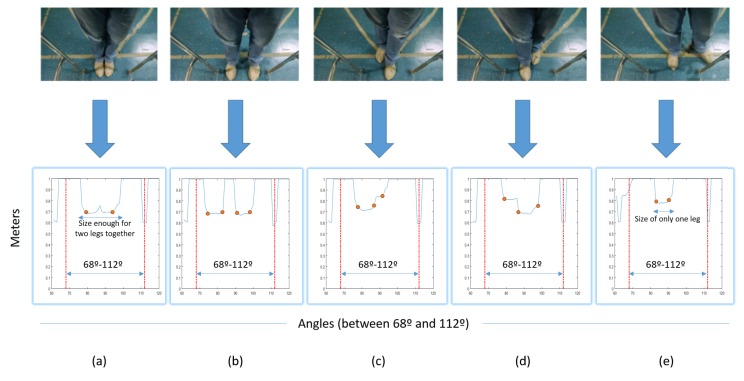
Diagram showing the operation of the leg detection algorithm. (**a**) Legs togheter - two transitions; (**b**) Legs separated - four transitions; (**c**) Three transitions - left leg closer to the laser sensor; (**d**) Three transitions - right leg closer to the laser sensor; (**e**) Only one leg detected.

There are five possible cases that are analyzed by the algorithm to find each human’s leg, in addition to estimate the human’s pose (legs’ position and orientation). The first case happens when there are less than two transitions or more than four transitions. In such cases, the algorithm ignores the frame and waits for the next laser scan. If there are more than 20 frames ignored consecutively, there is a safety rule that stops the walker. The other cases are when there are two, three or four transitions detected. Figure 6 shows examples of leg detection for: two transitions (a); four (b); three with the left leg closer to the laser (c); three with the right leg closer to the laser (d) and a case in which only one leg is detected; the other is outside the safe zone, and the laser sensor cannot detect it (e). The system can know this by analyzing the leg diameter in the space between two transitions. To be considered a leg, it should be bigger than the predefined value of 9 cm (after projection). The value 9 cm was taken based on the average horizontal line (frontal plane) of the leg at a 30-cm height, which is the laser height from the ground, of people that work in our laboratory. The dots in the graphics represent the variations, i.e., the transitions.

**Figure 7:**
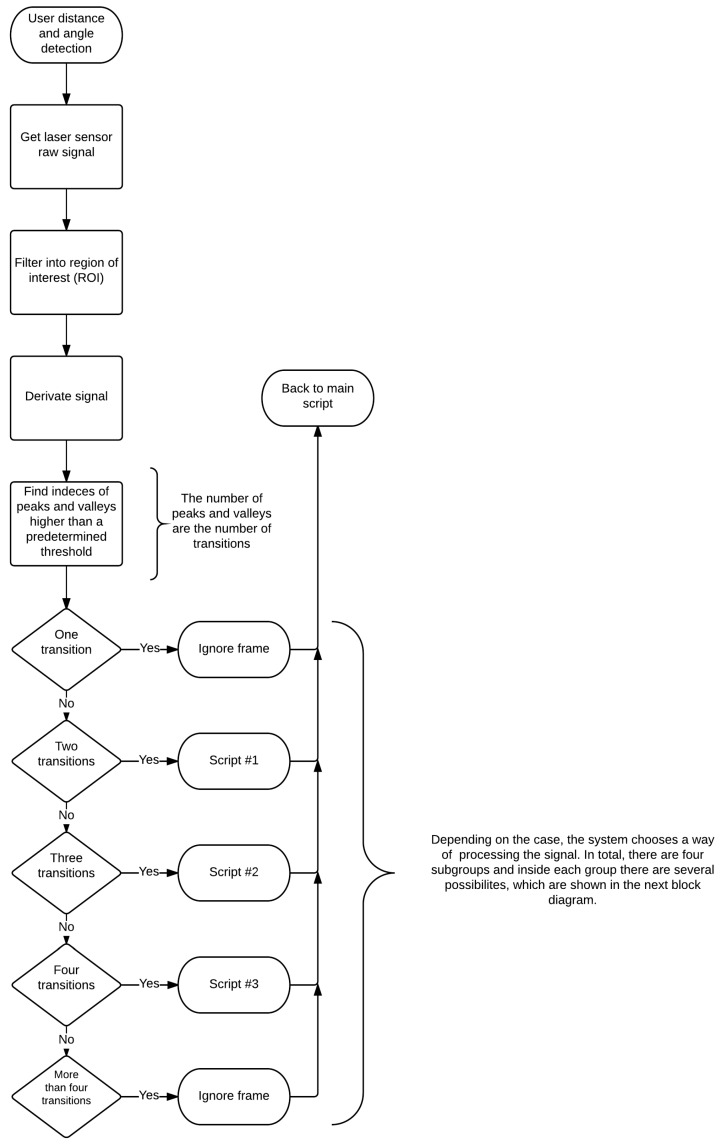
Flowchart used to infer the human position by finding where the legs are (main part).

**Figure 8:**
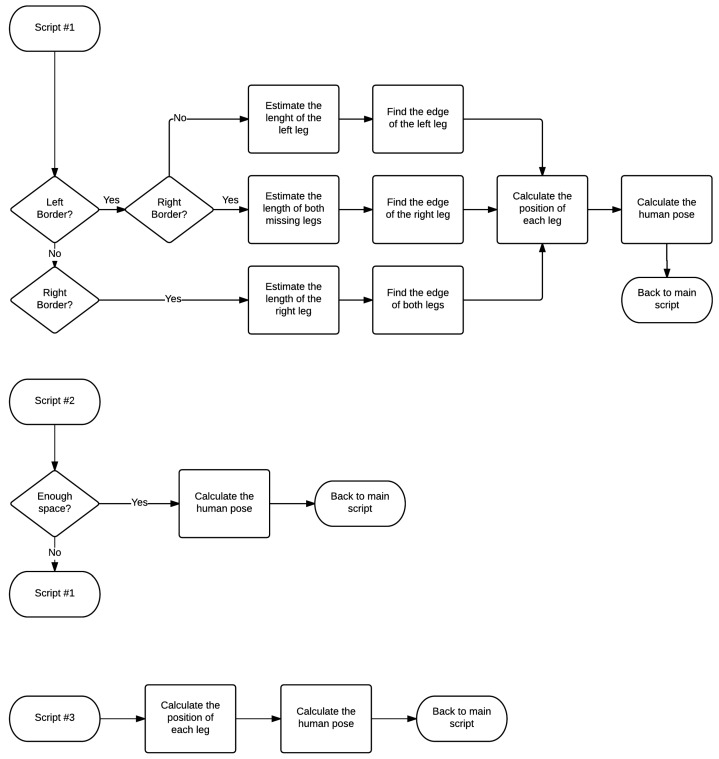
Details of the scripts for each kind of leg detection.

The flowcharts represented in Figure 7 show the decision tree used to determine where the user is, by analyzing the signals from the laser sensor for each case aforementioned. Figure 8 is an extension of Figure 7, which details how the number of transitions makes the algorithm behave.

In the case of the system finding less than two transitions, which means not detecting any leg or detecting just one leg, the system ignores the frame and increments the safety counter. If this counter reaches a maximum value, the walker stops. If there are only two transitions, the legs are probably in superposition, without significant difference between them regarding the distance from the laser sensor.

Figure 7 and Figure 8 show the algorithm used to make the system detect where the user’s legs are. First, the algorithm starts reading the laser sensor with its 180 angle range and a 30-m distance range. Second, it is necessary to crop the area into the region of interest, which is the area behind the walker. This area is defined by the angles between 68 and 112 and 1 m long. Everything outside this area is ignored.

Then, using this filtered signal, the algorithm computes its derivative and then finds where the major variations are, which are most probably the user legs. The transitions should be higher than a predefined threshold. This avoids the system obtaining noises or transitions, which could be misinterpreted as representing legs. With the information of the derivative of the laser signal, the algorithm finds the peaks and valleys, which indicate the transitions.

According to the number of transitions, distinct scripts are used to process the information. If there is only one transition, this laser reading is ignored and the robot waits for the next one. With only one transition, it is not possible to detect and define where the two legs are. The same behavior is adopted in the case of more than five transitions, since in this case, it is impossible to detect what transitions represent the legs.

For the other cases, it is possible to calculate the position of each leg. For two legs, the extreme position of the transitions is analyzed, and the person is considered to be in the middle between these points. Mathematically, this is represented by Equations (1) and (2).

(1) $$\begin{array}{c} L_{L}=T_{1}+\frac{\phi }{2} \end{array}$$

(2) $$\begin{array}{c} R_{L}=T_{2}-\frac{\phi }{2} \end{array}$$

where $L_{L}$ is the left leg and $R_{L}$ is the right leg. $T_{1}$ and $T_{2}$ are the first and second transitions (from left to right), respectively. The variable *ϕ* is related to the size of the leg projected in the distance of each transition.

In the case of the legs, there are two possibilities, which are either the left leg being closer to the laser or the right leg being closer to the sensor. To determine which leg is closer, the first and the last transition are analyzed. If the first transition ($T_{1}$) is closer to the sensor, this means the left leg is in front of the right leg. Otherwise (if $T_{3}$ is closer to the sensor), this means the right leg is in front of the left leg.

In the first case, the mathematical expression that would represent each leg position is given by Equations (3) and (4).

(3) $$\begin{array}{c} L_{L}=\frac{T_{1}+T_{2}}{2} \end{array}$$

(4) $$\begin{array}{c} R_{L}=T_{3}-\frac{\phi }{2} \end{array}$$

where $T_{3}$ is the third transition (from left to right).

On the other hand, the second case is given by the mathematical expressions shown in Equations (5) and (6).

(5) $$\begin{array}{c} L_{L}=T_{1}+\frac{\phi }{2} \end{array}$$

(6) $$\begin{array}{c} R_{L}=\frac{T_{2}+T_{3}}{2} \end{array}$$

In the case of four transitions, the leg angles are defined by Equations (7) and (8).

(7) $$\begin{array}{c} L_{L}=\frac{T_{1}+T_{2}}{2} \end{array}$$

(8) $$\begin{array}{c} R_{L}=\frac{T_{3}+T_{4}}{2} \end{array}$$

After detecting each leg position, the user position is estimated by taking the average position of both legs. The information given by $L_{L}$ and $R_{L}$, as well as the transitions are actually the indexes (angles) in the vector of the laser signal. To find the distance, it is necessary to look for the amplitude (distance, represented by variable *d*) given by those angles. Therefore, the user distance and angle from the walker are defined as in Equations (9) and (10).

(9) $$\begin{array}{c} d_{h}^{L}=\frac{d\left(L_{L}\right)+d\left(R_{L}\right)}{2} \end{array}$$

(10) $$\begin{array}{c} \varphi_{h}^{L}=\frac{L_{L}+R_{L}}{2} \end{array}$$

where $d_{h}^{L}$ is the distance from the human to the robot, $d(L_{L})$ is the distance of the left leg and $d(R_{L})$ is the distance of the right leg (Equation (9)). In Equation (10), $\varphi_{h}^{L}$, $L_{L}$ and $R_{L}$ are, respectively, the human orientation and his/her left and right leg orientation (angle). The superscript *L* means the laser axis, and the subscript *h* means the human/user.

#### 2.2.2. Human-Robot Kinematics

The human-robot interaction is given by a system that helps the user to move him/herself with the aid of the weight support and balance. In addition, different from other works that use inertial measurement units and/or force sensors, there are no sensors attached to the user’s body. All sensing is made by the robot, which is the follower in the formation. The laser sensor is used to verify where the user is, and in the case of obstacle detection, the ultrasound sensors present in the robot are also used. Therefore, in this work, a human-robot interaction is shown , in which the human does not need to wear any sensor, since all of the data needed are acquired by the robot and its sensors.

**Figure 9:**
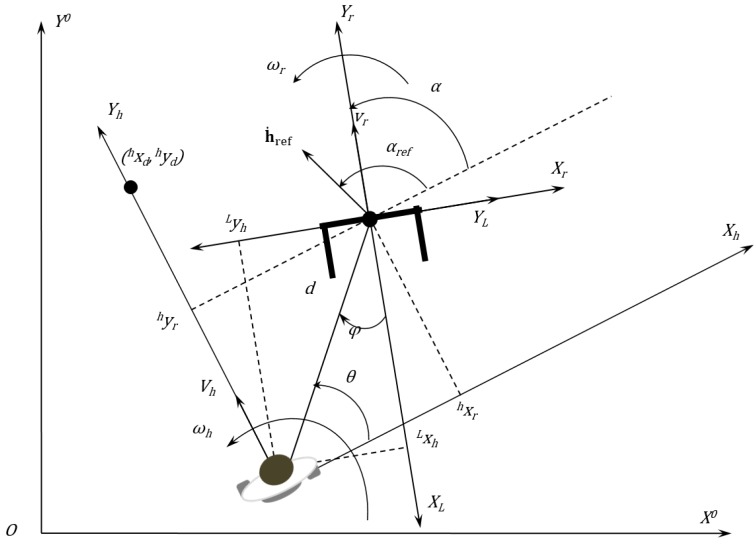
Diagram showing the human-robot interaction and the human, robot, laser sensor and absolute references.

The diagram of the human-robot interaction is depicted in Figure 9. This is the diagram used to generate the human-robot kinematics model and, further, the control laws. In the diagram depicted in Figure 9, it is possible to see the variables used by the controller algorithm to calculate the robot’s linear and angular speed to keep the formation. The variables presented in this picture are described in Table 2.

The mathematical model of the smart walker pose in the human reference is described in Equations (11)–(13). Pioneer 3-DX is a differential mobile robot with non-holonomic constraints.

(11) $$\begin{array}{ccc} & & {\dot{x}}_{r}^{h}=v_{r}^{0}\cdot \cos\alpha +\omega_{h}^{0}\cdot d\cdot \sin\theta \end{array}$$

(12) $$\begin{array}{ccc} & & {\dot{y}}_{r}^{h}=v_{r}^{0}\cdot \sin\alpha +\omega_{h}^{0}\cdot d\cdot \cos\theta -v_{h} \end{array}$$

(13) $$\begin{array}{ccc} & & \dot{\alpha }=\omega_{r}^{0}-\omega_{h}^{0} \end{array}$$

where $\dot{x}$ and $\dot{y}$ are the robot-human position variation due to his/her movements (linear speed $v_{h}$ and angular speed $w_{h}$) and *α* is the robot-human angle also related to his/her displacements. *θ* is the angle of the robot in the human reference, and *d* is the distance between the human and the robot. Velocities $v_{r}^{0}$ and $\omega_{r}^{0}$ are, respectively, the robot linear and angular speed.

In such a model, all variables with the index *h* are related to the user (human), while variables with index *r* are related to the robot (smart walker). The index *L* and 0 mean, respectively, the laser sensor and absolute references.

As shown in Equations (11)–(13), the absolute speeds of the human (both linear and angular) are needed to calculate the control actions. On the other hand, as shown in the diagram in Figure 4, the first step made by the controller after receiving the distance and angle from the laser sensor is to convert them into the Cartesian system using the laser sensor as the reference.

The following steps are used to find the linear and angular speed the robot should perform to keep the formation. Some of those steps can be visualized in Figure 4.

**Figure 10:**
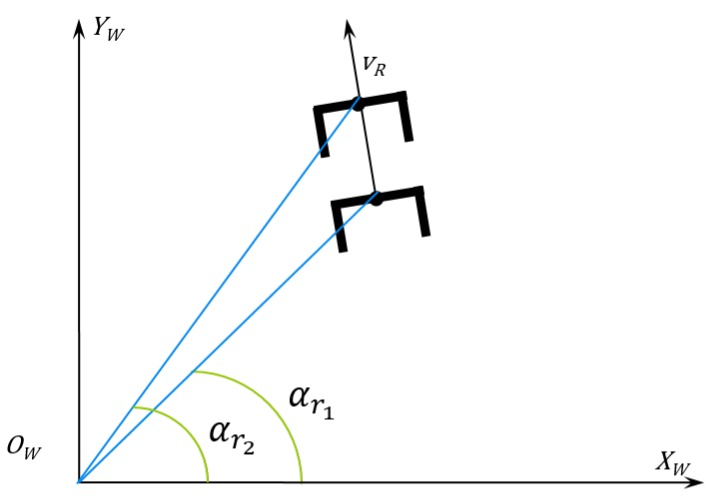
Variation of the robot in the absolute reference and calculation of the angular speed.

**Figure 11:**
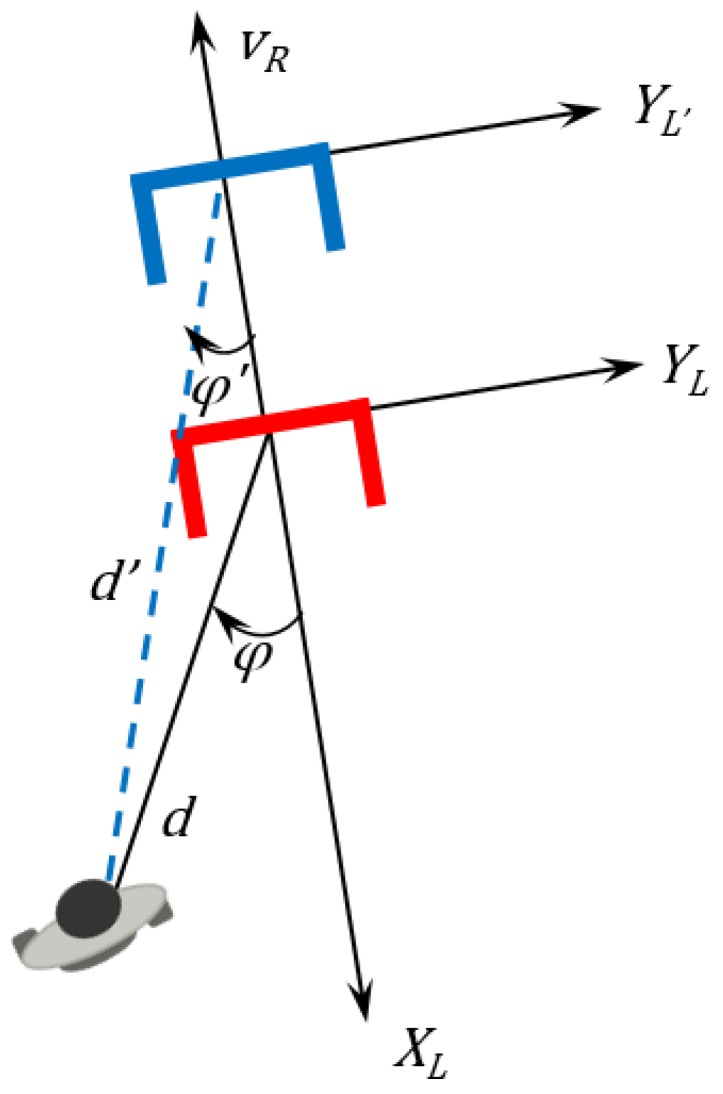
Projection of the previous position in the current time.

1. First, the coordinates are calculated in the Cartesian system (Equation (14)).
  (14) $$\left(\begin{array}{c} x_{h}^{L}\left(k\right)\\ y_{h}^{L}\left(k\right) \end{array}\right)=d\left(k\right)\left(\begin{array}{c} \cos\varphi (k)\\ \sin\varphi (k) \end{array}\right)$$
  where $x_{h}^{L}\left(k\right)$, $y_{h}^{L}\left(k\right)$ are the Cartesian coordinates with the laser sensor reference at the instant *k*. These coordinates are calculated using the distance and angle from the laser sensor (d(k) and φ(k)).
2. Second, after computing the human coordinates in the laser reference, it is necessary to measure the human linear and angular speed. To this end, it is necessary to calculate the robot angle variation, which is given by Equation (15). Figure 10 depicts the absolute angle robot variation.
  (15) $$\Delta \alpha_{r}\left(k\right)=\alpha_{r}\left(k\right)-\alpha_{r}\left(k-1\right)$$
  where $\alpha_{r}$ means the robot absolute angle, and $\Delta \alpha_{r}$ is its variation in two consecutive instants.
3. Following the control algorithm, this variation is used to calculate the rotation and translation transform matrices used to find the human previous location projected in the current one (Figure 11).

The projection shown in Figure 11 is represented in Equation (16).

(16) $${\left(\begin{array}{c} x_{h}^{L^{\prime }}\\ y_{h}^{L^{\prime }} \end{array}\right)}_{k-1|k}=\left(\begin{array}{cc} \cos(\Delta \alpha_{r}\left(k\right)) & \sin(\Delta \alpha_{r}\left(k\right))\\ -\sin(\Delta \alpha_{r}\left(k\right)) & \cos(\Delta \alpha_{r}\left(k\right)) \end{array}\right)\cdot {\left(\begin{array}{c} x_{h}^{L}\\ y_{h}^{L} \end{array}\right)}_{k-1}-\left(\begin{array}{c} v_{r}\cdot \Delta k\\ 0 \end{array}\right)$$

where:

- $x_{h}^{L}$ and $y_{h}^{L}$ are the human position in the laser reference at instant k−1.
- $x_{h}^{L^{\prime }}$ and $y_{h}^{L^{\prime }}$ are the previous item position projected into the current instant *k*.
- $v_{r}$ is the robot linear speed.
- Δk is the sample time between two consecutive instants of time (in the figure, the time needed for the walker to move from $Y_{L}$, which is supposed to be in the instant k−1, to $Y_{L^{\prime }}$, which is supposed to be in instant *k*, which is the current instant.
- Subscript k−1|k means the positions in instant k−1 projected into instant *k*.

This information is useful to calculate the speed of the human using a single reference, which is *k*.

4. Once the human’s previous location projected on the current robot reference and the current user location are available, it is possible to compute the human linear and angular speeds. The human speed in this case is the same for the robot reference and the absolute reference. This occurs because it is a variation, and the robot displacement in the absolute reference was taken into account in the last part of Equation (16).

**Figure 12:**
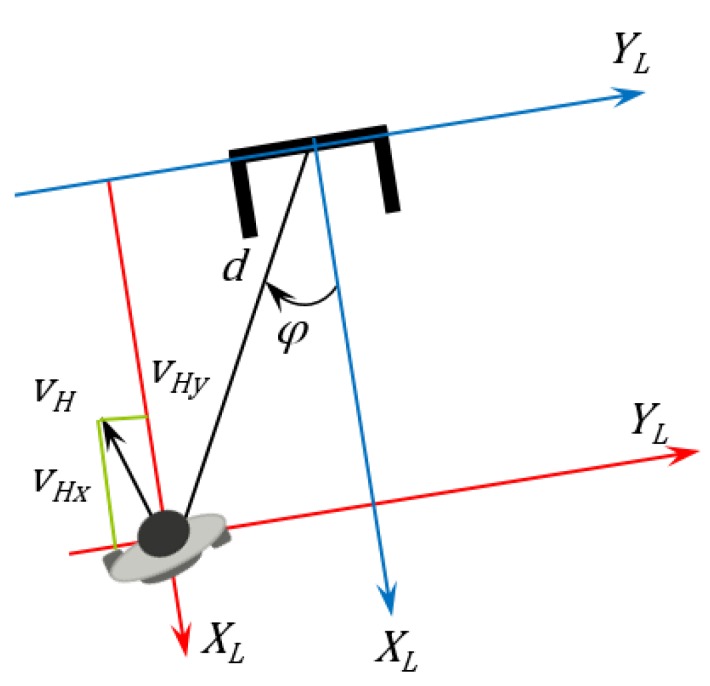
Human speed vector calculation.

Figure 12 shows the user speed calculation based on the robot movement. It is considered that the human did not move.

Therefore, it is possible to calculate the absolute human linear speed in both axes (*x* and *y*), in addition to the module of the speed, as shown in Equations (17)–(19), respectively.

(17) $$\begin{array}{c} v_{h_{x}}\left(k\right)=\frac{x_{h}^{L}\left(k\right)-x_{h}^{L}\left(k-1|k\right)}{\Delta k} \end{array}$$

(18) $$\begin{array}{c} v_{h_{y}}\left(k\right)=\frac{y_{h}^{L}\left(k\right)-y_{h}^{L}\left(k-1|k\right)}{\Delta k} \end{array}$$

(19) $$\begin{array}{c} v_{h}\left(k\right)=\sqrt{v_{h_{x}}^{2}\left(k\right)+v_{h_{y}}^{2}\left(k\right)} \end{array}$$

This velocity value is independent of the reference system that is being used.

**Figure 13:**
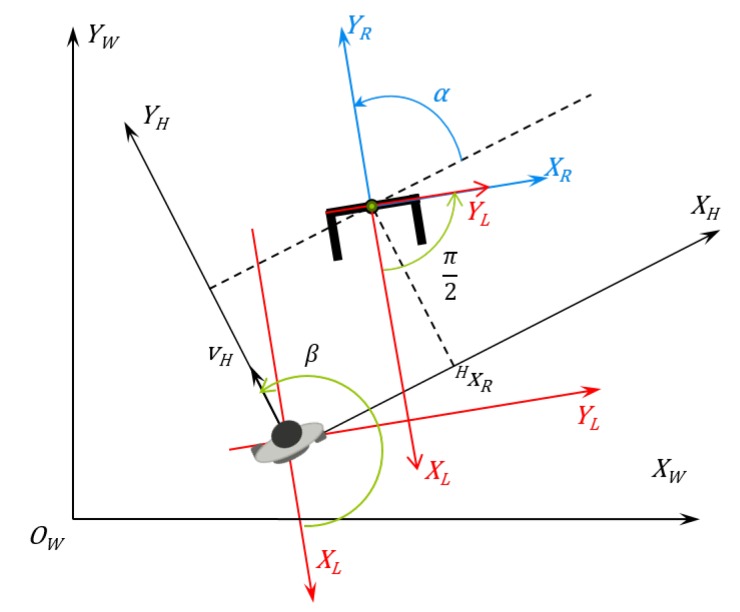
Calculation of the angle of the human in the laser reference.

5. The next step relies on computing the human orientation in the robot reference, as shown in Figure 13.

This information is necessary to find the human angular speed, which is exactly the angle between the two speed components of the human speed, given by Equation (20).

(20) $$\beta \left(k\right)=\arctan\frac{y_{h}^{L}\left(k\right)-y_{h}^{L}\left(k-1|k\right)}{x_{h}^{L}\left(k\right)-x_{h}^{L}\left(k-1|k\right)}$$

where *β* is the human speed vector angle (angle between the components of the human speed).

**Figure 14:**
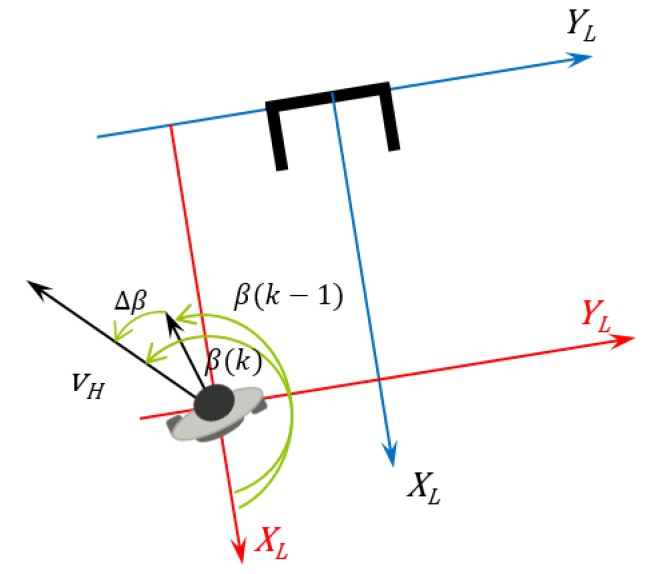
User angular speed by *β*-angle variation.

**Figure 15:**
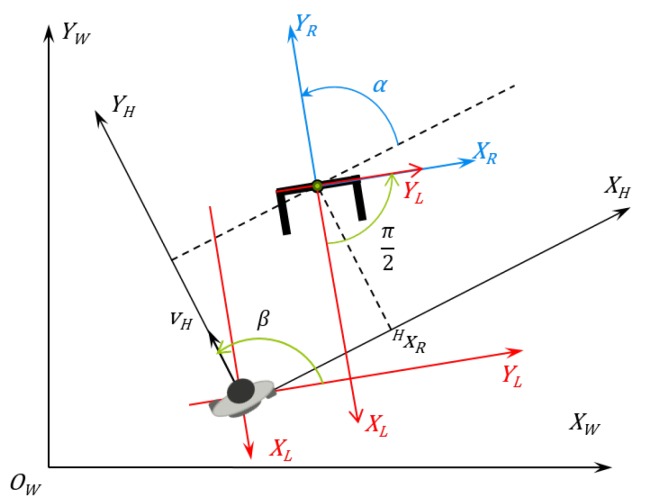
Conversion from the laser reference to the robot reference. Note that the *β* angle goes from the $X_{H}$ axis up to the $Y_{L}$ axis. Since it goes clockwise, it is negative.

**Figure 16:**
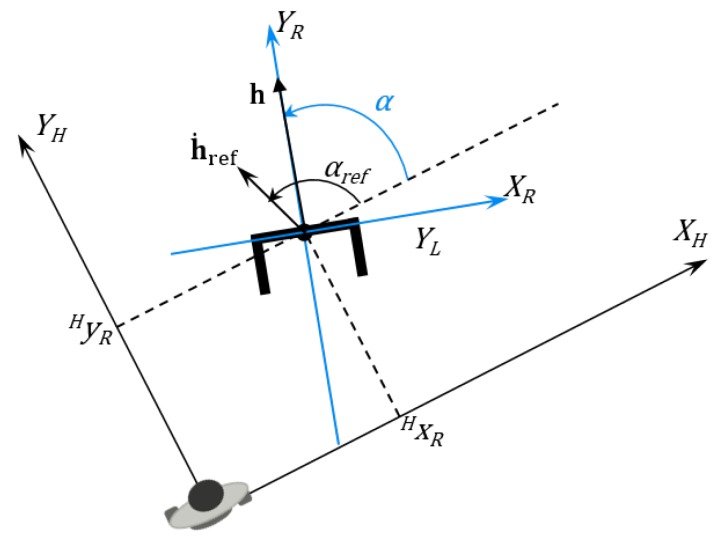
Robot position projected into the human reference.

6. Using the variation of the angle *β*, it is possible to find the human angular speed on the robot reference. The robot angular speed $\omega_{r}$ is previously provided by the robot encoders. Figure 14 shows how the angle variation affects the user angular speed.
  (21) $$\omega_{h}\left(k\right)=\frac{\beta (k)-\beta (k-1)}{\Delta k}+\omega_{r}\left(k\right)$$
  where $\omega_{h}$ and $\omega_{r}$ are the absolute speeds of the human and the robot.
7. From the human orientation in the robot’s reference, it is possible to find the robot’s orientation in the human reference, through Equation (22). The angle between the laser reference and the robot reference is $\frac{\pi }{2}$ (assuming counterclockwise positive), as shown in Figure 15.
  (22) $$\alpha \left(k\right)=-\left(\beta \left(k\right)+\frac{\pi }{2}\right)$$
8. Following the calculation of the control actions, the next step is to find the displacement vector **T**, which converts the robot’s reference into the user’s reference.
  (23) $$T\left(k\right)=\left(\begin{array}{cc} \cos(\frac{\pi }{2}) & \sin(\frac{\pi }{2})\\ -\sin(\frac{\pi }{2}) & \cos(\frac{\pi }{2}) \end{array}\right)\cdot \left(\begin{array}{c} x_{h}^{L}\left(k\right)\\ y_{h}^{L}\left(k\right) \end{array}\right)$$
9. Finally, it is possible to compute the robot position in the human reference, as shown in Equation (24) and represented in Figure 16.
  (24) $$\left(\begin{array}{c} x_{r}^{h}\left(k\right)\\ y_{r}^{h}\left(k\right) \end{array}\right)=\left(\begin{array}{cc} \cos(-\alpha_{r}\left(k\right)+\frac{\pi }{2}) & \sin(-\alpha_{r}\left(k\right)+\frac{\pi }{2})\\ -\sin(-\alpha_{r}\left(k\right)+\frac{\pi }{2}) & \cos(-\alpha_{r}\left(k\right)\frac{\pi }{2}) \end{array}\right)\cdot \left(\begin{array}{c} 0-T(k) \end{array}\right)$$
  where $x_{r}^{h}$ and $y_{r}^{h}$ are the human position in the robot reference.
10. To define the control laws, it is important to define first the vector **h**, which contains the robot position in the human reference, shown in Equation (25). Figure 16 details the desired vector $h_{d}$ and the current vector *h*.
  (25) $$h=\left[\begin{array}{c} x_{r}^{h}\\ y_{r}^{h} \end{array}\right]$$
11. It is also essential to determine which are the desired values, given by the $h_{d}$ vector, represented in Equation (26).
  (26) $$h_{d}=\left[\begin{array}{c} x_{r}^{h}{|}_{d}\\ y_{r}^{h}{|}_{d} \end{array}\right]$$
  where the variables with subscript *d* indicate the desired values, i.e., the set-points of the robot position vector and each one of its components.
12. The error vector is given by Equation (27).
  (27) $$\tilde{h}=h-h_{d}$$
13. By using the inverse kinematic model, the speed reference vector can be calculated as in Equation (28)
  (28) $${\dot{h}}_{ref}=-K\tilde{h}-{\dot{h}}_{r}^{h}$$
  where **K** is a control gain, and ${\dot{h}}_{r}^{h}$ (shown in Equation (28)) refers to the human contribution to the whole system movement, given by:
  (29) $${\dot{h}}_{r}^{h}=\left[\begin{array}{c} \omega_{h}\cdot d\cdot \sin\theta \\ -\omega_{h}\cdot d\cos\theta -v_{h} \end{array}\right]$$
  where *θ* refers to the human angle in the absolute reference.
14. Finally, the control laws are computed, as described in Equations (30) and (31).
  (30) $$\begin{array}{ccc} & & v_{c}=\left|{\dot{h}}_{ref}\right|\cos\dot{\alpha } \end{array}$$
  (31) $$\begin{array}{ccc} & & \omega_{c}=k_{\omega }\tilde{\alpha }+{\dot{\alpha }}_{ref}+\omega_{h}, \end{array}$$
  where $v_{c}$ and $\omega_{c}$ are the controller outputs. Thus, these are reference speeds the controller sends to the robot.

The kinematic controller is presented in Equations (30) and (31). Even though the dynamic model can be affected by the user’s weight, the usually slow speeds during robot operation make it irrelevant to consider the dynamic effects. Therefore, a pure kinematic controller is sufficient to keep the user distance and angle, and the dynamic model alterations due to the user and structure weight can be ignored. Besides, part of the weight is not supported by the robot, but the walker frame and the user’s legs.

#### 2.2.3. Control Stability Proof

The stability proof of the controller is made by analyzing the whole system with the direct Lyapunov method. Considering the state vector as the error vector, the positive definite function of Equation (32) is taken:

(32) $$V=\frac{1}{2}\tilde{h}{\tilde{h}}^{T}$$

The derivative function of Equation (32) is shown in Equation (33).

(33) $$\dot{V}=\tilde{h}{\dot{\tilde{h}}}^{T}$$

By substituting ${\dot{\tilde{h}}}^{T}={\dot{h}}_{ref}=-K\tilde{h}$, it is possible to find that this derivative function is negative definite, as shown in Equation (34), thus concluding the asymptotic stability at the equilibrium point of zero error.

(34) $$\dot{V}=-\tilde{h}K\tilde{h}<0,\forall \tilde{h}\neq 0,\forall K>0$$

Similarly, we can prove that the robot’s orientation converges to zero by taking the candidate function (Equation (35)).

(35) $$V=\frac{1}{2}{\tilde{\alpha }}^{2}>0$$

Deriving Equation (35), we find:

(36) $$\dot{V}=\tilde{\alpha }\dot{\tilde{\alpha }}$$

To prove Equation (36) is negative definite, it is necessary to close the loop and isolate the term $\dot{\tilde{\alpha }}$. Therefore, considering that the controller speed equals the robot speed, which means $\omega_{c}$ of Equation (31) equals $\omega_{r}^{0}$ of Equation (13), we can obtain Equation (38) by computing this information into Equation (13).

(37) $$\begin{array}{c} \dot{\alpha }=\omega_{r}^{0}-\omega_{h}^{0} \end{array}$$

(38) $$\begin{array}{c} \dot{\alpha }=\omega_{c}-\omega_{h} \end{array}$$

(39) $$\begin{array}{c} \dot{\alpha }=k_{\omega }\tilde{\alpha }+{\dot{\alpha }}_{ref}+\omega_{h}-\omega_{h} \end{array}$$

(40) $$\begin{array}{c} 0=k_{\omega }\tilde{\alpha }+{\dot{\alpha }}_{ref}-\dot{\alpha } \end{array}$$

(41) $$\begin{array}{c} 0=k_{\omega }\underset{-\dot{\tilde{\alpha }}}{\underset{︸}{{\dot{\alpha }}_{ref}-\dot{\tilde{\alpha }}}} \end{array}$$

(42) $$\begin{array}{c} \dot{\tilde{\alpha }}=-k_{\omega }\tilde{\alpha } \end{array}$$

Now, by replacing Equation (42) in Equation (36), Equation (43) can be obtained, thus proving that $\dot{V}$ is negative definite.

(43) $$\dot{V}=-K_{w}{\tilde{\alpha }}^{2}<0$$

#### 2.2.4. Safety Rules

Safety rules are a special part of the algorithm, apart from the controller, which analyzes whether the controller’s output is safe or not. Normally, the controller’s outputs are executed; however, there are some special situations that may require a safety supervisor. Examples of these situations are when the laser sensor only detects one leg, which may imply that the user is losing his or her balance and may fall. Table 3 shows the safety rules used in this work and when they are applicable. Thus, the safety supervisor can change the controller’s output, in order to guarantee human safety.

## 3. Obstacle Avoidance

**Figure 17:**
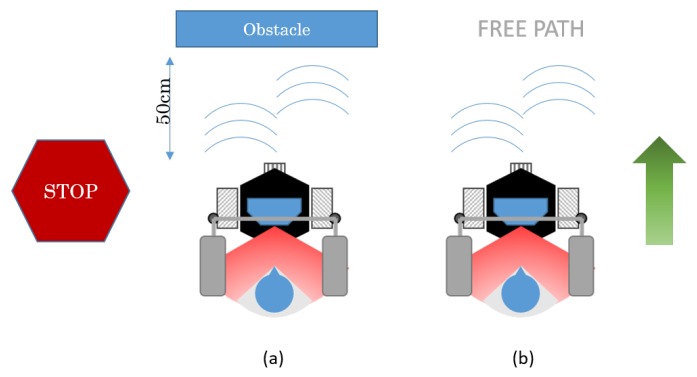
Ultrasound sensors are used to avoid collision. In (**a**), it stops moving to avoid the obstacle; in (**b**), it keeps moving, since there is no obstacle.

The robot is equipped with ultrasound sensors that can be used to avoid obstacles. If the obstacle avoidance is turned on, the robot stops if there is an object or person within a 50-cm distance in front of the robot. Figure 17 shows how the ultrasounds act in the case of finding an obstacle in front of the robot.

## 4. Experiments

Experiments were performed in order to validate the controller and safety rules by applying them in real situations. This is a proof-of-concept application; therefore, the goal is to prove that the system can work, and it was not tested with people suffering from disabilities.

### 4.1. Straight Line

**Figure 18:**
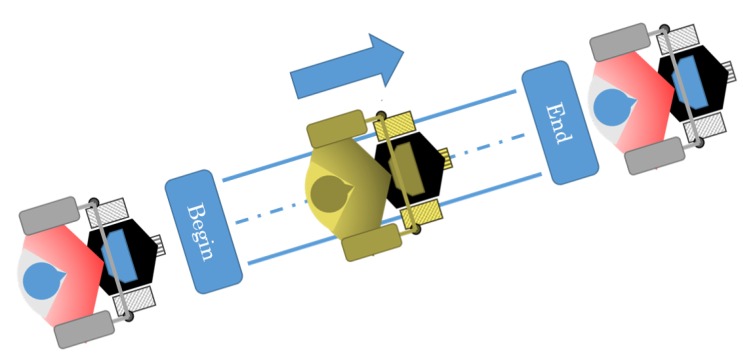
Straight path where the user guided the robot. The human walked through that path, and the robot helped him to perform such an action. The human guides the robot, not the opposite.

**Figure 19:**
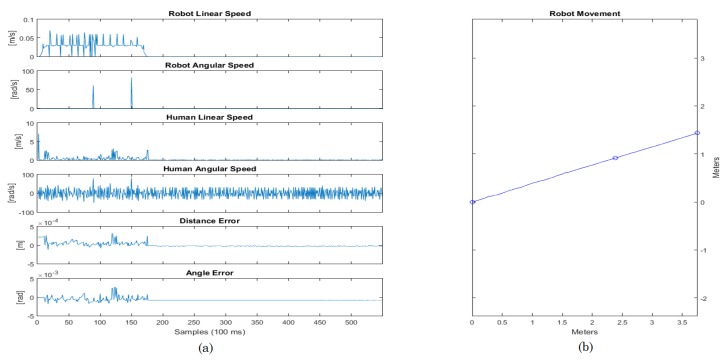
Results for the straight path. (**a**) Graphical data about the experiment; (**b**) Graphical path of the experiment.

In the first experiment set, the user was asked to walk helped by the smart walker in a 10-m straight path, three times. The user goes from the start point until the finish point and then returns back to the start point. In the first instance, there is no obstacle in the line the user goes through. In the second instance, a wood board is placed, which represents an obstacle that should be detected by the ultrasound sensor and brake the walker, following Safety Rule #5. The path and movements are described in Figure 18. As can be seen in the results (Figure 19), the errors in the distance and angle converge towards zero, and since it is a straight line, there is almost no angular speed.

The error tends to zero, but it does not converge due to the movement of the user. During the walking, the human-robot distance changes not only due to the robot movement, but also due to the human movement. Therefore, the controller always tends to force the error towards zero, but when the user moves, it changes the distance again, making the error different than zero. Since the speed is limited, the robot cannot act so quickly in order to always maintain the error at zero. However, it stays bounded, which shows that the controller is acting while the user walks.

In the case of the straight line experiment, the angle error was −0.04 rad, while the distance error was 0.0009 m.

In order to validate the obstacle detection algorithm, which is Safety Rule #5, the ultrasound sensors are monitored, and the walker stops immediately if the robot finds an obstacle within 50 cm from the robot. In the beginning of this experiment, there is an obstacle, and when the robot achieves 50 cm from the obstacle, it stops. Then, the obstacle is removed, and the walker can move forward again.

**Figure 20:**
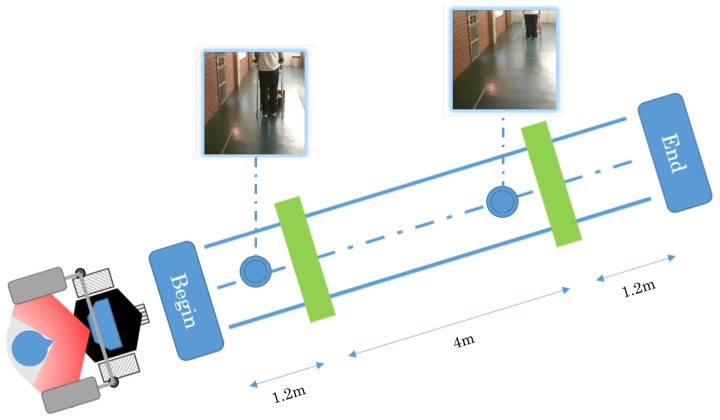
Diagram of the path with obstacles.

**Figure 21:**
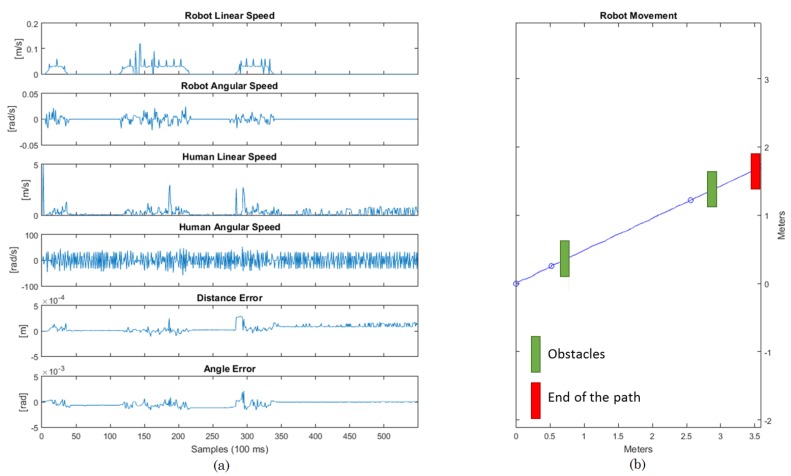
Results for the straight path with obstacles. (**a**) Graphical data about the experiment; (**b**) Graphical path of the experiment, showing the obstacles and the end of the path.

Figure 20 shows some photos of the experiment and the diagram of the path with the obstacles. Every time the obstacle was put in front of the smart walker, it stopped, and once it was removed, the device started moving again. The results of this experiment are shown in Figure 21.

Similarly to the previous case, the errors stay bounded. When the walker approximates the obstacle, it stops in order not to collide with it. Only when the obstacle is removed does it allow the user to walk again. In this experiment, the angle error was −0.02 rad, while the distance error was 0.05 m.

### 4.2. Lemniscate Path

**Figure 22:**
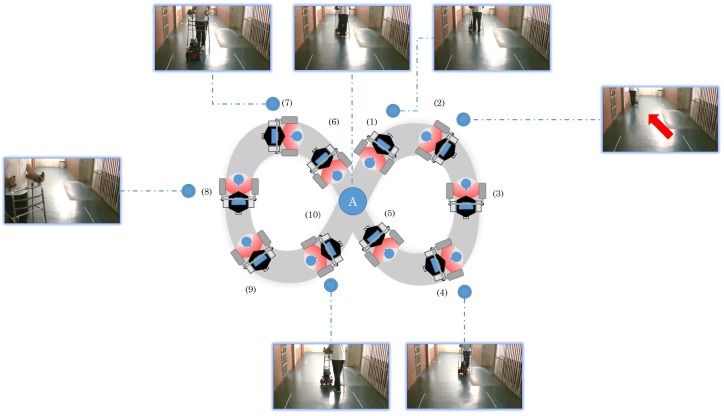
Lemniscate curve followed in the second set of experiments (with photos).

The second experiment was conducted with the walker following a Lemniscate curve path (which is typically used to validate robot performance). In the first instance, there is no obstacle, while in the second instance, the walker should brake before colliding with the obstacle. The user started the curve at Point “A” and then walked following the whole curve, returning to the same point, as shown in Figure 22.

Mathematically, this Lemniscate curve is represented by Equations (44) and (45).

(44) $$\begin{array}{ccc} & & x\left(t\right)=a\cdot \frac{cos(t)}{sin^{2}\left(t\right)+1} \end{array}$$

(45) $$\begin{array}{ccc} & & y\left(t\right)=b\cdot \frac{sin(t)\cdot cos(t)}{sin^{2}\left(t\right)+1} \end{array}$$

where *a* and *b* are the constants defining the length in each axis.

**Figure 23:**
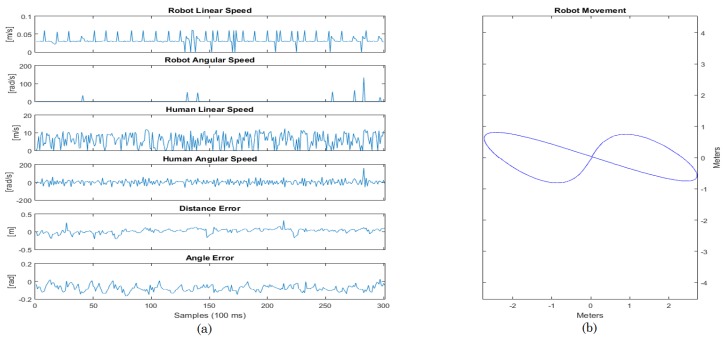
Results for the Lemniscate curve. (**a**) Graphical data about the experiment; (**b**) Graphical path of the experiment.

The Lemniscate curve followed by the robot can be viewed in Figure 23. The error varies through time, but always tends to zero.

The shape generated by the robot odometry in Figure 23 is relative to the Lemniscate curve. The shape does not fit completely in the curve shown due to two reasons: (1) the human guided the robot, and therefore, he did not walk exactly on the Lemniscate curve; (2) there are some odometry errors due to sliding in addition to other errors that naturally accumulate throughout time when using odometry. The control error tends to zero, with means the distance error is 0.02 m and the mean angle error equals −0.08 rad. It is important to emphasize that the error varies with the human movement, i.e., it will always be changing while the human moves, but the controller makes it go towards zero throughout time.

After collecting the data from the experiments, some results, a discussion and conclusions can be made, which are shown in Section 5 and Section 6.

## 5. Discussion

The results of the experiments show that the controller maintained stability and helped the user in different paths, including complex curves, such as the Lemniscate one. In addition, the safety rules were functional when necessary.

The graphs in Figure 23 show that in abrupt changes, there were slight increases of the errors, but they quickly went towards zero, due to the effect of the controller. Additionally, in the cases when the safety rules were needed, the robot responded in a suitable way, detecting the obstacle and braking the smart walker.

Still, regarding the safety rules, when only one leg was detected, the smart walker was stopped. This is an important issue addressed here, as the one leg detection may be the cause of the falling of the user.

The results of this research show that the controller kept the set-point distance and angle. Additionally, the safety rules also have been activated as expected.

This shows that the idea behind the controller, sensors and actuators together with the mechanical structure, i.e, the whole structure of the smart walker, can be a useful tool for mobility and rehabilitation and also may be used in clinics for such purposes.

## 6. Conclusions

This work presented a new controller and a safety supervisor to guide a smart walker with no sensors attached to the user. Additionally, it was shown how these concepts were applied in a modified walker frame attached to a mobile robot, which has a laser sensor to detect the user’s legs. In addition, ultrasound sensors on-board this robot allowed braking the smart walker to avoid the collision with obstacles.

The algorithm used to detect the user’s legs was suitable for this application, and the whole system integration was tested with two different kinds of experiments, involving obstacles and free paths (without obstacles).

The results were found satisfactory, and this controller, together with the safety rules, is a potential tool for helping people both for mobility and for physical rehabilitation, since the controller proved to guarantee safe walking. The safety rules behaved as expected, offering the user a safe experience, needed both in mobility and rehabilitation.

## Figures and Tables

**Table 1:** Smart walkers’ controllers and sensors.

Smart Walker	Sensors	Controllers
RT Walker [37]	Force/moment sensing and encoders	Several algorithm controllers for motion (obstacle avoidance, path following, among others)
GUIDO Smart Walker [29]	Laser sensor, force sensors, switches, sonar and encoders	Shared control approach
PAM-AID [38]	Ultrasound and laser sensors	Algorithmic controller
PAMM [36]	Health sensors, external sensors, encoders, force sensors, among others	Admittance-based controller
JARoW [34]	Infrared sensors	Algorithmic controllers
iWalker [30]	RFID, encoders and external sensors	Algorithmic controllers
UFES’ Smart Walker [22]	IMUs, laser sensor and force sensor	Force and inverse kinematics controllers
Our device	Laser sensor, encoders and ultrasound	Formation-based controller

**Table 2:** Variables in Figure 9.

Variable	Details	Unit
$v_{h}$	Human linear speed in the absolute axis	m/s
$\omega_{h}$	Human angular speed in the absolute axis	rad/s
*d*	Human-robot distance	m
*θ*	Robot angle in the human reference	rad
*φ*	Human angle in the robot reference	rad
$x^{L}$ and $y^{L}$	Laser sensor longitudinal and transversal axis	m
$x^{H}$ and $y^{H}$	Human longitudinal and transversal axis	m
$x^{R}$ and $y^{R}$	Robot longitudinal and transversal axis	m
$x_{h}^{L}$ and $y_{h}^{L}$	Human position in the laser sensor reference	m
$x_{h}^{R}$ and $y_{h}^{R}$	Human position in the robot reference	m
$x_{R}^{h}$ and $y_{R}^{h}$	Robot position in the human reference	m
${\dot{h}}_{ref}$	Speed vector the robot should follow to keep the formation	m/s
*α*	Robot orientation in the human reference	rad
$\alpha_{ref}$	Set-point orientation the robot should achieve to keep the formation	rad
$v_{r}$	Robot linear speed	m/s
$\omega_{r}$	Robot angular speed	m/s
*β*	Human orientation in the robot reference	rad
$v_{h_{x}}$	Human linear speed in the transversal axis	m/s
$v_{h_{y}}$	Human linear speed in the longitudinal axis	m/s
*k*	Sample time	0.1 s (100 ms)
k−1	Previous sample time	0.1 s (100 ms)
k−1|k	Positions and angles of instant (k−1) projected into instant (*k*)	0.1 s (100 ms)

**Table 3:** Safety rules for the smart walker.

Situation	Action	Notes
No legs/only one leg detected, several times sequentially	Increase the counter	There is a counter that increases each time both legs are not detected. If this counter reaches the limit number, the walker stops immediately. The limit number can be defined inside the code. If the leg is detected before the counter reaches the limit, the counter is zeroed.
Counter exceeded limit	Brake the robot	If the counter reaches the maximum limit, it brakes the robot and stops its movement.
High speed	Limit speed	This safety rule is applicable for both linear and angular speeds.
Backwards movement	Brake the robot	Braking the robot in this case means the speed will be set up to zero.
Obstacle (detected by ultrasound sensors)	Brake the robot	The robot is stopped while the obstacle is not removed from the path.

## Abbreviations

The following abbreviations are used in this manuscript:

LD: Leg detection
LMS: Laser measurement systems
LRF: Laser range finder
PID: Proportional-integral-derivative
ROI: Region of interest
